# Initial Angiotensin Receptor Blocker Response in Young Marfan Patients Decreases After 3 Years of Treatment

**DOI:** 10.1007/s00246-021-02761-4

**Published:** 2021-11-10

**Authors:** Christiane Pees, Julian Heno, Ina Michel-Behnke

**Affiliations:** Division of Pediatric Cardiology, Department of Pediatric Cardiology, Pediatric Heart Center Vienna, University Children’s Hospital, Pediatric and Young Adult Marfan Syndrome Outpatient Clinic Vienna, Medical University Vienna, Währinger Gürtel 18-20, 1090 Vienna, Austria

**Keywords:** Marfan syndrome, Children, Losartan, Longitudinal follow-up

## Abstract

Marfan syndrome is caused by mutations of the fibrillin-1 gene, which weakens the connective tissue integrity. Since 2003, bioavailability regulations of TGF-ß through fibrillin alterations have been presumed of being the culprit mechanisms for aortic aneurysm development. We present the analysis of our single-center Marfan children and adolescents cohort to assess the influence of age, sex, degree of cardiovascular involvement and dosage on losartan effectivity. This prospective longitudinal registered echocardiographical investigation (EudraCT 2009-016139-36) of 49 patients with an average follow-up of 72 months focused on aortic root *z*-scores, elasticity, and yearly progression rates. The 33 patients under medication with losartan showed an aortic root *z*-score reduction during the first 36 months compared to 22 patients without medication presenting constant mild progression. Yet, results diminished under losartan thereafter, adding up to similar progressions over 72 months in both groups (0.07 ± 0.10/year versus 0.04 ± 0.11/year). Although male patients exhibited higher root *z*-scores, progression with and without medication was comparable to females and not age-dependent. In conclusion, losartan evoked a significant aortic root *z*-score regression in young Marfan patients over the first 3 years, but this effect mitigated thereafter. The initial improvement concurred with ameliorated elasticity; lower stiffness levels predicted better clinical outcome, but likewise only up to 36 months. Sex differences in dilatation severity were observed but neither age nor sex had significant influence on progression rates. Losartan dosages were gradually increased in more severely affected patients and provided an equal rate of root progression over 72 months in comparison to patients under losartan treatment with lesser baseline dilatation severity.

## Introduction

Marfan syndrome (MFS), a monogenetic disorder, is caused by pathogenic variants of the fibrillin-1 gene FBN1 [1]. Fibrillin-1 is a major component of the omnipresent connective tissue, which mutations predominantly affect cardiovascular, ocular and skeletal systems. Pathogenic variants were primarily thought to merely weaken the structural integrity of the extracellular matrix (ECM), where fibrillin, incorporated into microfibrils, provides a scaffold for elastin [2]. In 2003, Neptune proposed a second role for fibrillin-1 regarding the bioavailability of TGF-ß, showing that FBN1 alterations increase TGF-ß [3]. This was presumed the culprit mechanism for aortic aneurysms in thoracic aneurysm disorders like MFS [4]. Elevated TGF-ß levels in blood and connective tissue were measured in several genetically engineered aneurysm mouse models [5], and in human MFS aortic tissue [6]. The groundbreaking study of Habashi et al. in 2006 showed the normalization of aneurysms after administration of TGF-ß neutralizing antibodies as well as losartan, an Angiotensin-II-type-1-receptor-blocker (ARB): The TGF-ß hyperactivity was attenuated in Fbn1^C1039G/+^-mice, harboring a typical aortic disease-causing mutation [7]. Since then, further investigations have been performed in order to validate these results and to fully comprehend the pathological pathways [8, 9]. But clinical trials in MFS children are sobering: While the first open-label administration of losartan add-on to ß-blocker in highly affected children restrained aortic growth significantly [10], multi-center trials did not prove superiority of losartan over ß-blocker [11]. Dual therapy showed conflicting results, at its best slightly reducing but not normalizing excessive aortic growth [12].

For the presented study, we particularly assessed the influence of age, sex, cardiovascular involvement, losartan dosages and follow-up durations on losartan treatment achievements in a relatively homogenous group of MFS children and adolescents. We compared and analyzed the data of our patients with and without losartan treatment, which are all regularly followed up by a single investigator.

## Methods

The study protocol was approved by the Vienna University Hospital committee on human research institutional review board (462/2009) and registered in the European Union Drug Regulating Authorities Clinical Trials database (EudraCT 2009-016139-36). Written informed consent was mandatory and obtained from all patients and/or their relatives. This longitudinal prospective echocardiographic investigation of all children and adolescents with clinically diagnosed MFS from the Pediatric Marfan Clinic Vienna presents data of those with follow-up durations of a minimum of 36 months in the time frame from 2008 until 2019. Of the 69 patients diagnosed according to the revised Ghent criteria [13], 66 were validated genetically [14]. Prophylactic treatment with losartan was offered to all patients. In those with advanced aortic involvement [aortic root (AoR) *z*-score > 3], therapy was strongly recommended and well-accepted by the families and patients [15]. Treatment postponement wishes by the patient or their family were tolerated in patients with lesser cardiac involvement under continuous surveillance, especially if compliance to medical treatment was doubted. In patients receiving losartan, treatment was started with 0.2 mg/kg BID and doubled weekly, up to the highest tolerated dosage (tiredness, dizziness) in the first 3 months (1–2 mg/kg/day, max 150 mg/day). Further medical adaptation to body growth was performed at the regular check-ups and additionally if the patients experienced significant aortic *z*-score increase. Add-on of beta-blocker were discussed with the families in this situation as well.

Inclusion criteria of this survey were: (1) MFS diagnosis by Ghent criteria, (2) follow-up of minimal 36 months, (3) adherence to annual check-ups, (4) compliant medication intake with adequate adaptation during growth.

Exclusion criteria were: (1) AoR-replacement operation prior or during the first 36 months, (2) other connective tissue disorders, (3) pregnancy.

Patients’ data such as date of birth, MFS symptoms and genetic results were registered at baseline. Aortic diameters, corresponding normalized values, elasticity results, current age, weight, height, BSA, blood pressure were collected prospectively at annual check-ups. All echocardiographic data were carried out by a single investigator (CP): Diameters of the aortic valve, AoR, sino-tubular junction and ascending aorta were measured inner-edge to inner-edge in parasternal long-axis view using a VividE9 GE-Medical device (Horton, Norway). Measurements were normalized using BSA and corresponding *z*-score values were obtained applying the calculator www.parameterz.com by choosing the reference of Warren et al. [16], whereby *z*-scores ≥ 2 indicated dilation. AoR elasticity was measured in parasternal long-axis M-Mode. Offline stiffness and distensibility calculations with mean systolic and diastolic diameters were performed as previously described [17] using simultaneously measured blood pressures (Philips SureSignsVS2, Andover, USA).

Data analysis was performed with IBM-Statistics, version 26.0 (SPSS, IBM, Chicago). Kolmogorov–Smirnov tests were used to test for parametricity. For continuous variables, Student’s *t*-test was used, for non-parametric parameters Kruskal–Wallis or Mann–Whitney-*U* computations were applied. Correlation structures were tested using Pearson’s and Spearman’s correlation, as applicable. For longitudinal value depiction, LOESS regression was deployed. Data is given as two-sided *p*-values, considered statistically significant if *p* < 0.05.

## Results

**Fig. 1:**
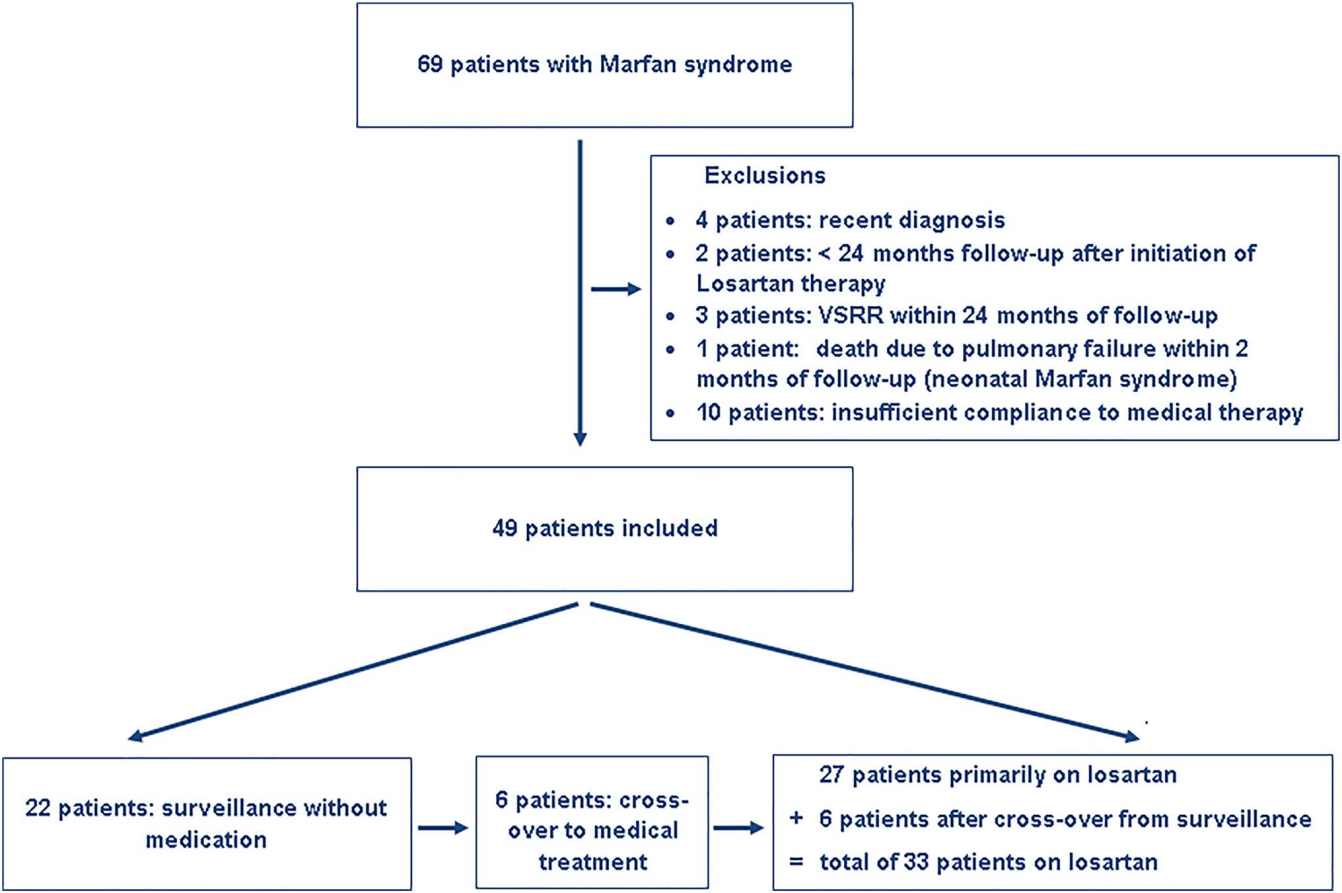
Patients’ recruitment. VSRR: valve sparing root replacement operation

**Fig. 2:**
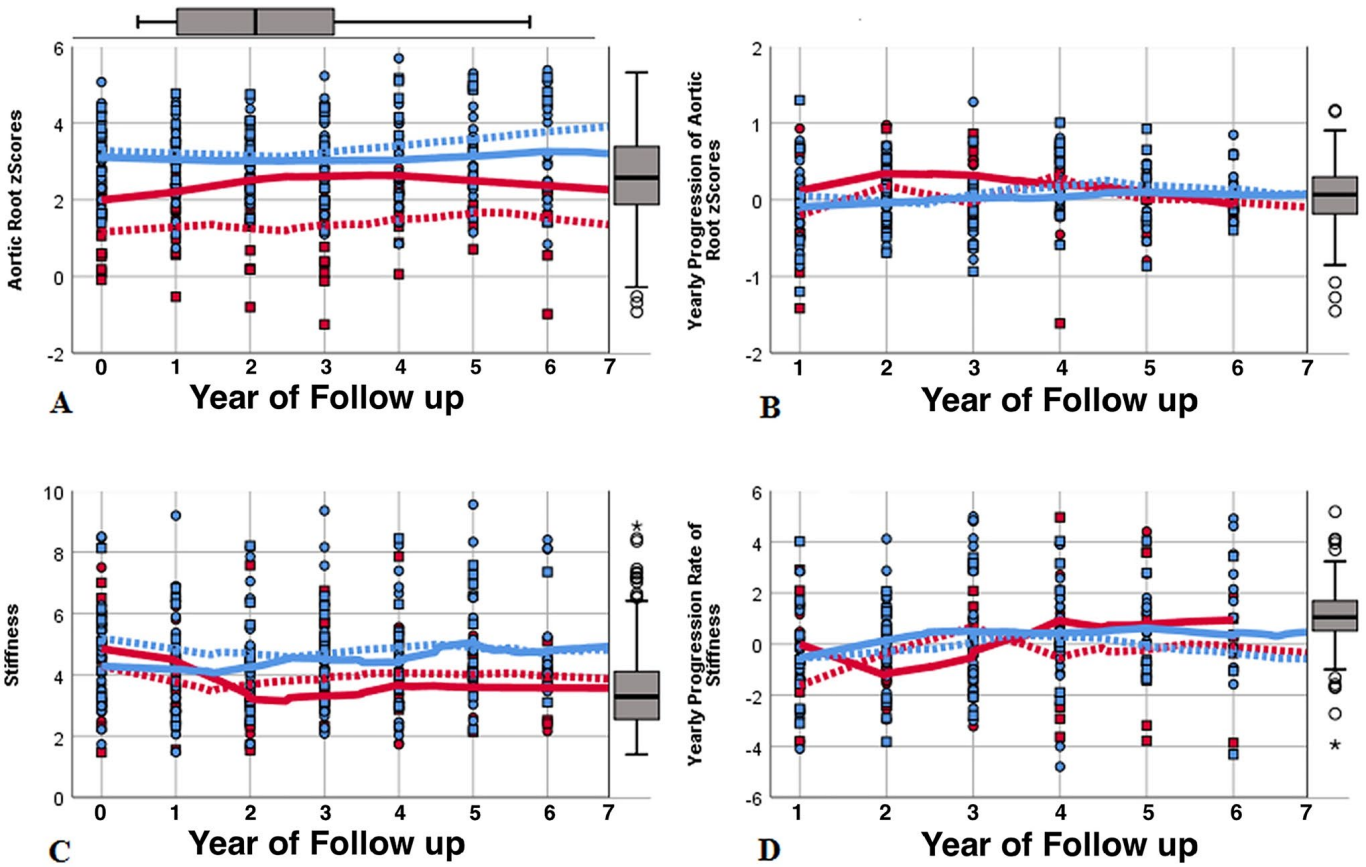
Individual *z*-scores of aortic root measurements and progression rates (**a**, **b**) as well as stiffness and stiffness progression rates (**c**, **d**) at baseline and at yearly follow-ups, calculated over 72 months, are depicted, with added LOESS regression curves. Patients without medication (red) showed lower aortic *z*-scores (**a**) but no differences in stiffness values (**c**) compared to patients receiving medication (blue) during the entire follow-up. A slight regression of aortic *z*-scores with losartan during the first 3 years compared to patients without medication was noticed. This course reversed thereafter, displaying only mild but equal progression rates of aortic *z*-scores in both patient subgroups (with and without medication) between examinations 4–7 years of follow-up (**b**). This effect was mirrored by both sexes, but males (circles, solid lines) showed higher aortic *z*-scores compared to females (squares, dashed lines) (**a**). Nearly equal elasticity levels were measured (**c**) but with a slight but non-significant deterioration in annual progression of stiffness over 72 months in males (**d**). Box plots show mean and quartiles of the respective variables

**Fig. 3:**
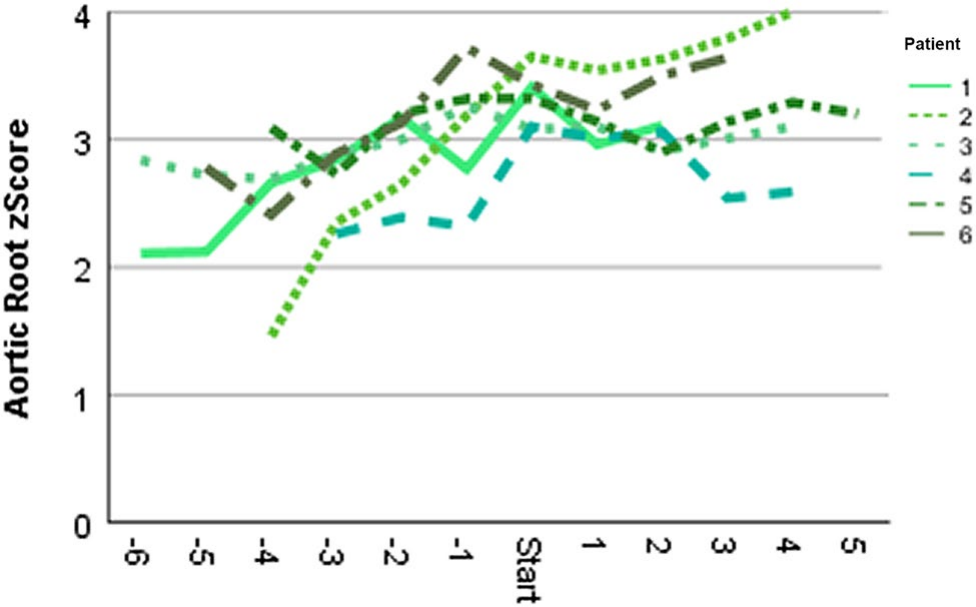
Depiction of annual aortic root *z*-scores of six patients primarily followed up without medication and then started on losartan (start), thereafter presenting nearly stagnant aortic root progression

Patients’ selection is depicted in Fig. 1, baseline characteristics are presented in Table 1. Follow-up duration was 6.6 years on average (range 3–11 years). MFS patients receiving losartan were significantly older, and hence taller and heavier. As expected, this group presented with significantly greater aortic dimensions and *z*-scores due to treatment decision options in the less affected patients. Blood pressure and elasticity values did not differ. Significantly higher AoR *z*-scores were found in the 27 males compared to the 22 females (2.72 ± 1.04 versus 2.00 ± 1.40; *p* = 0.04), but there were no sex differences regarding age, elasticity, and blood pressure (age: *p* = 0.928; stiffness: *p* = 0.99; systolic: *p* = 0.66; diastolic blood pressure: *p* = 0.50). During the first 36 follow-up months, patients on losartan treatment exhibited a regression of AoR *z*-scores, but presented with a significant progression thereafter over the following 3 years, with no similar changes noticeable in the patients without medication presenting with constant mild progression throughout the time period (Table 1). To prevent bias from different follow-up durations, we repeated this analysis with data of patients completing the entire observational period only (*n* = 19 under medication versus *n* = 11 without losartan), but gained equivalent outcomes. The individual annual AoR *z*-scores and progression rates are depicted in Fig. 2a and b. No sex influence in annual AoR *z*-score progressions calculated over 72 months was detected, neither in general (males: 0.08 ± 0.11/year; females: 0.05 ± 0.11/year; *p* = 0.44), nor in the losartan treatment subgroup (males: 0.05 ± 0.09/year, *n* = 23; females: 0.11 ± 0.11/year, *n* = 10; *p* = 0.227): Both sexes showed slight regressions with losartan during the first 36 months (− 0.03 ± 0.13/year and − 0.05 ± 0.24/year; *p* = 0.782), but the effect reversed equally thereafter (0.15 ± 0.14/year and 0.16 ± 0.06/year; *p* = 0.87). Six of our patients without medication, including one female, exhibited a significant increase in annual AoR *z*-scores of mean 0.27 ± 0.22, therefore, losartan treatment was discussed again and started thereafter, initiating a significant inhibition of aortic growth with an aortic *z*-score regression of − 0.05 ± 0.21/year over the following 36–60 months (*p* = 0.028; Fig. 3). No alterations in elasticity due to losartan treatment were detectable in this 6 patients (data not shown).

**Table 1:** Baseline patient characteristics and echocardiographic follow-up measurements

	Marfan patients with losartan treatment (*n* = 33)	Marfan patients without medication (*n* = 22)	*p*
Age (years)	10.8 ± 5.9	6.6 ± 4.7	**0.006**
Weight (kg)	38.8 ± 21.0	24.9 ± 16.7	**0.009**
Weight *z*-score	− 0.20 ± 1.3	− 0.33 ± 1.27	0.715
Height (cm)	152.9 ± 33.3	123.0 ± 33.8	**0.002**
Height *z*-score	1.95 ± 1.29	1.02 ± 0.99	**0.004**
BSA (m^2^)	1.25 ± 0.46	0.91 ± 0.40	**0.005**
Systolic blood pressure (mmHg)	102 ± 13	100 ± 12	0.628
Diastolic blood pressure (mmHg)	60 ± 7	60 ± 7	0.840
Aortic valve diameter (mm)	22.2 ± 4.6	16.8 ± 3.5	** < 0.001**
Aortic valve *z*-score	2.80 ± 0.81	1.35 ± 0.84	** < 0.001**
Aortic root diameter (mm)	30.9 ± 6.6	22.6 ± 5.2	** < 0.001**
Aortic root *z*-score	3.15 ± 0.93	1.53 ± 0.93	** < 0.001**
Sinotubular junction diameter (mm)	23.9 ± 5.1	17.1 ± 4.0	** < 0.001**
Sinotubular junction z-score	2.48 ± 1.27	1.03 ± 1.07	** < 0.001**
Ascending aortic diameter (mm)	22.1 ± 4.4	15.8 ± 3.7	** < 0.001**
Ascending aortic *z*-score	1.21 ± 1.17	-0.58 ± 1.31	** < 0.001**
Stiffness	5.19 ± 2.39	4.56 ± 1.63	0.267
Distensibility of aortic diameter (cm^2^ × dynes^−1^ × 10^–6^)	6.10 ± 3.06	6.74 ± 4.50	0.563
Distensibility of aortic area (kPa^−1^ × 10^–3^)	49.0 ± 25.3	55.7 ± 37.9	0.482
Follow-up			
Progression of aortic root *z*-score/year (0–36 months)	− 0.04 ± 0.17	0.09 ± 0.24	**0.030**
Progression of aortic root *z*-score/year (36–72 months)	0.15 ± 0.13	− 0.02 ± 0.17	**0.034**
Progression of aortic root *z*-score/year (0–72 months)	0.07 ± 0.10	0.04 ± 0.11	0.468
Progression of stiffness/year (0–36 months)	− 0.17 ± 0.85	− 0.16 ± 0.70	0.991
Progression of stiffness/year (36–72 months)	0.47 ± 1.01	0.01 ± 0.55	0.247
Progression of stiffness/year (0–72 months)	0.13 ± 0.61	− 0.19 ± 0.38	0.188
Follow-up duration mean and range (months)	83.6 (36–132)	57.7 (36–96)	0.271

Statistically significant differences are given in bold

Data presented as mean ± standard deviation except follow-up which is presented as mean and (range)

The devolution of elasticity is depicted in Fig. 2c and d: Although no differences were noted at baseline, stiffness showed a mild annual regression during the first 3 years followed by a progression thereafter with and without losartan (Table 1). Subdivided by sex, the annual progression of stiffness over 72 months exhibited a slight but non-significant deterioration in males (0.03 ± 0.54/year) compared to a mild amelioration in females (− 0.15 ± 0.57/year; *p* = 0.414) in all patients, as well as in the subgroup under losartan treatment (males: 0.19 ± 0.53/year; females: − 0.09 ± 0.92/year; *p* = 0.424).

**Fig. 4:**
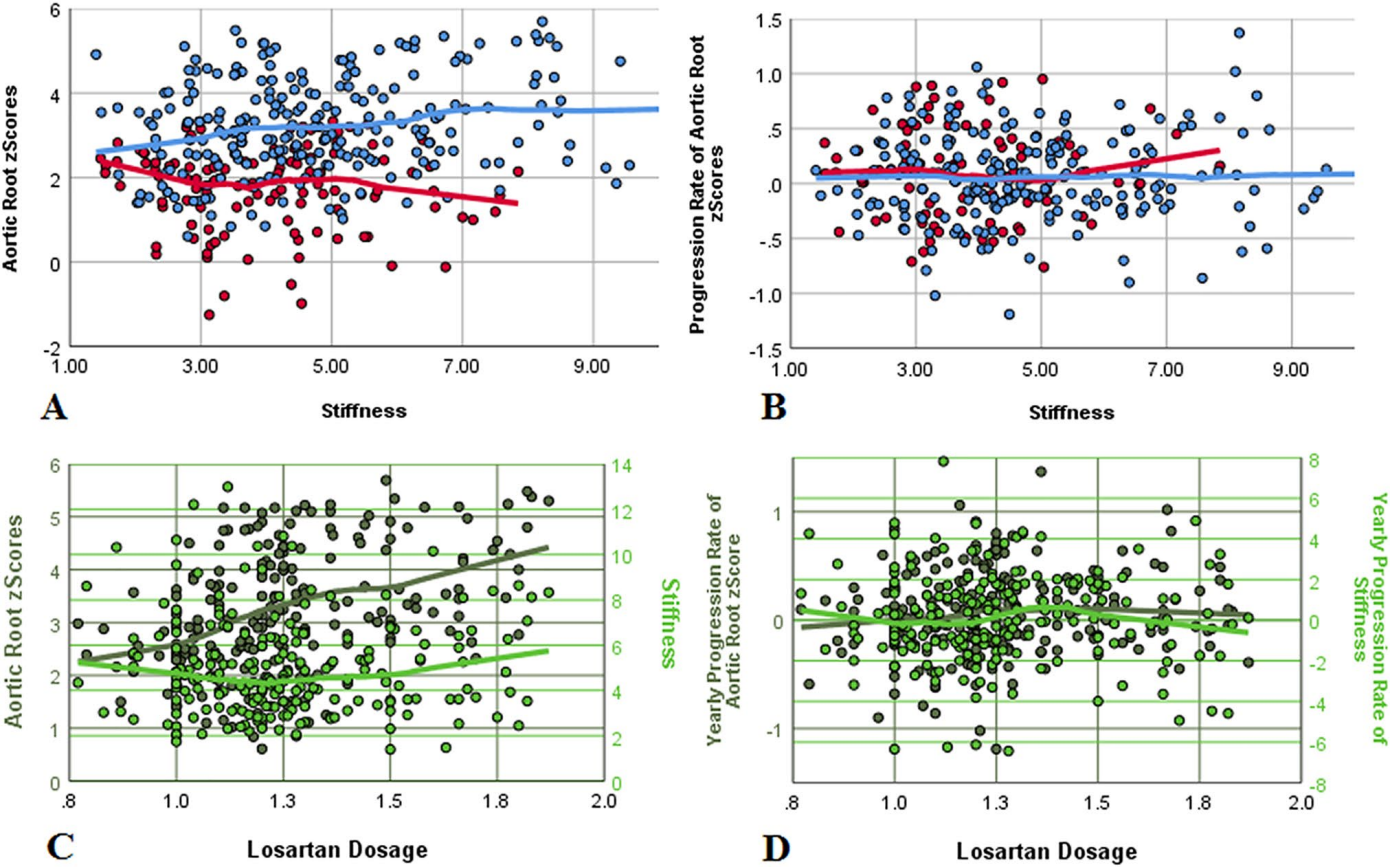
Individual *z*-scores of aortic root measurements (**a**) as well as progression rates (**b**) at baseline and at yearly follow-ups showed a significant correlation between individual aortic *z*-scores and stiffness measurements in patients without medication (red) and under losartan treatment (blue) by added LOESS regression curves (**a**), but stiffness did not show an influence on *z*-score progression in both subgroups (**b**). Plotting aortic *z*-scores (dark green) and stiffness levels (light green) against the individual losartan dosages showed that more affected patients received higher up-titrated losartan dosages (**c**), thereby keeping *z*-score progressions independent of baseline levels constant (**d**) with no correlation of losartan dosage with aortic stiffness (**c**) or stiffness progression (**d**)

A significant correlation between AoR *z*-scores and stiffness was revealed (*r* = 0.222; *p* < 0.001) when plotting annual AoR against the corresponding stiffness (Fig. 4a), but stiffness did not show an influence on *z*-score progression (Fig. 4b). By calculating percentiles of our stiffness values (10th < 2.49, median 4.22, 90th > 7.39), we attempted a more detailed examination of this correlation in all patients: Those with a stiffness > 90th percentile (*n* = 6, 2 females) showed a tendentially higher mean AoR progression rate compared to patients with lower stiffness values (0.10 ± 0.07/year versus 0.05 ± 0.10/year; *p* = 0.09). Of note, age and baseline AoR *z*-scores did not differ. In patients treated with losartan, again a stiffness value < 90th percentile at baseline predicted a statistically lower progression of the AoR *z*-scores during the first 36 months (− 0.04 ± 0.17/year versus 0.05 ± 0.15/year; *n* = 5; *p* = 0.041). Progression rates thereafter as well as on average over the entire 72 months period were indistinguishable.

Mean losartan dosage was 1.26 ± 0.23 mg/kg/day with no detectable sex differences (males: 1.27 ± 0.25 mg/kg/day; females: 1.23 ± 0.18 mg/kg/days; *p* = 0.15). Three patients received add-on atenolol therapy due to significantly higher AoR *z*-scores (4.11 ± 0.41 on dual-, 2.99 ± 1.02 on mono- and 1.53 ± 0.92 without therapy at baseline; *p* < 0.001). Correlation equations proved a highly significant correlation of AoR baseline *z*-scores with losartan dosages, showing that more affected patients received higher up-titrated losartan dosages (*r* = 0.407, *p* < 0.001; Fig. 4c), thereby keeping AoR progressions constant, independent of baseline AoR *z*-scores (Fig. 4d). No correlation of losartan dosage with aortic stiffness (Fig. 4c) or stiffness progression (Fig. 4d) was detectable.

To investigate the progression rates in relation to baseline AoR dilation severity in greater detail, we divided our treatment group into AoR severity subgroups with (A) *z*-score < 3; (B) *z*-scores ≥ 3–4, and (C) *z*-scores ≥ 4. By comparing these subgroups no distinctions in age, body size, blood pressure or elasticity nor its progression were detected. AoR *z*-score progression was comparable with A: − 0.03 ± 0.11/year, B: − 0.05 ± 0.22/year and C: − 0.01 ± 0.09/year (*p* = 0.979) during months 0–36 and 0.11 ± 0.17/year, 0.20 ± 0.08/year and 0.17 ± 0.08/year during months 36–72, respectively (*p* = 0.236). Again, only losartan dosages were significantly different with 1.17 ± 0.21, 1.29 ± 0.22 and 1.37 ± 0.24 mg/kg/day, respectively (*p* < 0.001).

**Fig. 5:**
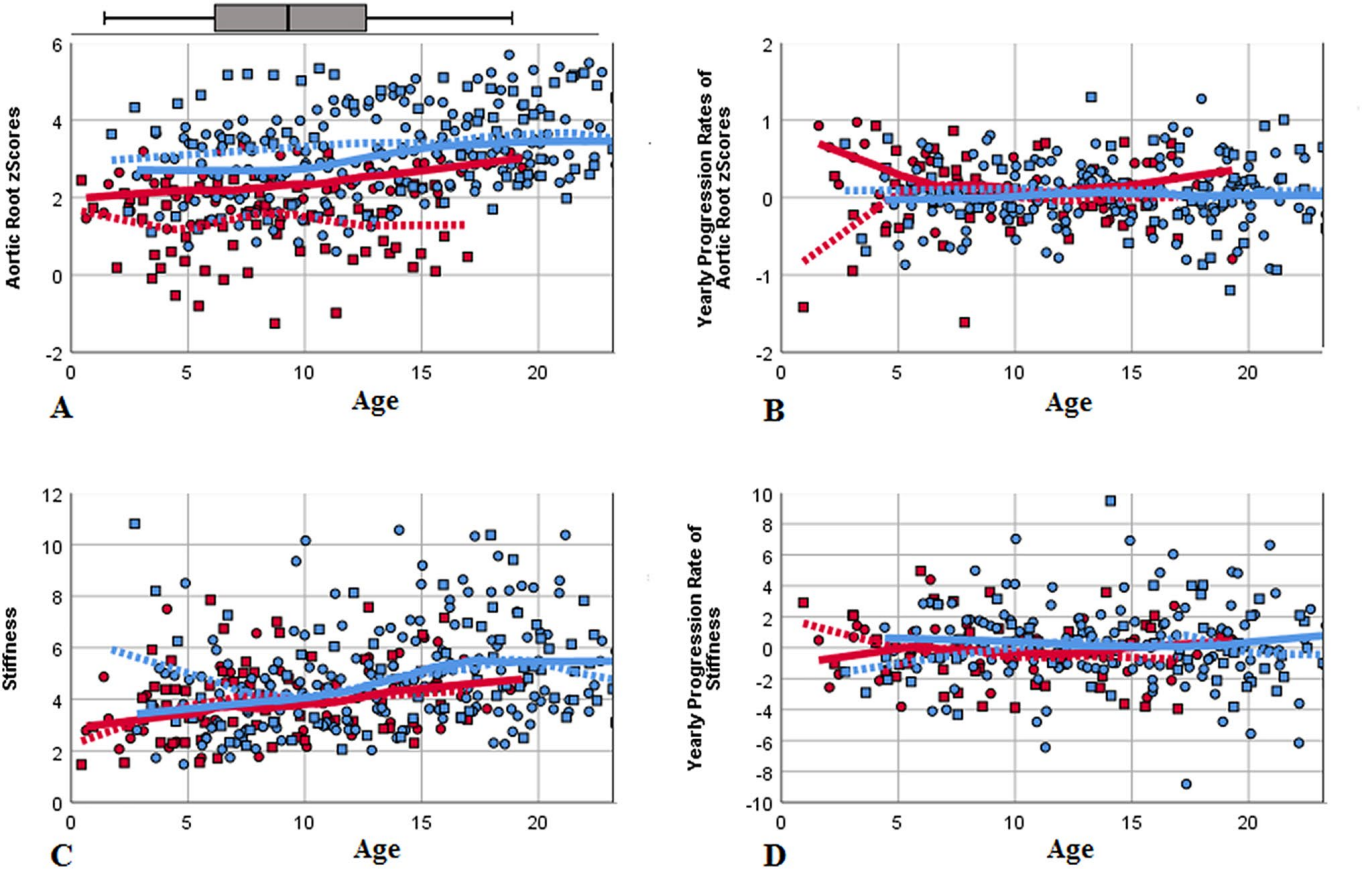
Age dependency on aortic progression was ruled out by plotting the individual *z*-scores of aortic root measurements (**a**) and progression rates (**b**) against the patients’ ages at examination with added LOESS regression curves. Stiffness showed a mild but not significant increase with age (**c**) but the corresponding progression rates not (**d**). Again, red depicts patients without medication and blue patients under losartan treatment with females as squares and dashed lines and males as circles with solid lines. The box plot shows mean and quartiles of age

Age dependency on AoR progression was ruled out by plotting the individual AoR *z*-scores (Fig. 5a) and stiffness values (Fig. 5c) and their corresponding annual progressions (Fig. 5b, d) against the individual age at time of follow-up. A significant deterioration of AoR *z*-scores during puberty could not be revealed. Although AoR *z*-scores correlated highly significantly with age (*r* = 0.397; *p* < 0.001), the individual progression of *z*-scores over the follow-up period did not (*r* = − 0.033; *p* = 0.575). Comparably, stiffness itself (but not annual stiffness progression) correlated with age (*r* = 0.329; *p* < 0.001, *r* = − 0.014; *p* = 0.81).

## Discussion

This longitudinal investigation of 49 children and adolescents with MFS demonstrates an effective and significant regression of AoR *z*-scores in patients under losartan treatment over 36 months in contrast to those without medication, although the latter group was younger and exhibited lesser AoR baseline dilatation. This observation is comparable to earlier studies [11, 15]. The regression capability of losartan could also be illustrated in 6 cross-over patients, in whom AoR progression was reverted by losartan, which mirrors the first publication of highly affected MFS children [10]. Surprisingly, the demonstrated regression of aortic *z*-scores in patients under losartan treatment mitigated during the following 36 months to a progression of aortic *z*-scores, in comparison to the steady low *z*-score progression in the surveillance group throughout the 72 months period. This extenuating effect of losartan after 36 months was somehow unexpected, thus we tried to identify possible influencing factors that could explain the weakening effect of prolonged losartan treatment. In a recent study by Teixido-Tura et al. [18], the long-term outcome of 128 MFS patients (with 45 below the age of 18) under losartan or atenolol treatment for a mean of 6.7 ± 1.5 years was presented in referring to the results gained in the preceding LOAT trial [19]. In the LOAT trial, a comparable AoR diameter increase in both intervention groups during the first 3 years of the LOAT trial was measured by MRI investigation with 1.1 mm/3 years (= 0.37 mm/year) in the losartan and 1.4 mm/3 years (= 0.47 mm/year) in the atenolol group, with a mean *z*-score regression of − 0.4 and − 0.1, respectively (*p* = 0.193). In the open-label extension of this study [18], a similar AoR increase of 0.4 mm/year in both subgroups was found. But the diameter increase was presented only as a difference of 2 MRI measurements with an interval between the measurements of 5.9 ± 2 years. Therefore, our echocardiographic data illustrates a more precise evaluation of the yearly progression rates.

Since fibrillin-1 mutations weaken the ECM integrity, elasticity measurements seem to play a major role in detecting structural tissue disorders [20]. Besides building an elastin scaffold, fibrillin-1 connects vascular smooth muscle cells to the aortic elastic lamellae, giving the tissue a tense but distensible structure [21]. ECM stabilization is further provided by proteoglycans, generating osmotic pressure due to negative charges, attracting mobile counterions and hereby causing a swelling pressure, thus supporting the microfibril-cell connections essential for the mechanosensing function of aortic smooth muscle cells [22]. Many different syndromic and non-syndromic single gene alterations predisposing to ascending aortic aneurysms encode one of the mentioned elements of the aortic media structure [21]—supporting the assumption of constant biomechanical forces on structurally weaker tissue as a principle cause of aneurysm formation. Although in our cohort, the indexed baseline AoR diameters—markers for the disruption of aortic integrity in MFS—were significantly different in the treatment and non-treatment groups, elasticity of the ascending aorta did not differ. Mean elasticity levels were only slightly stiffer and less distensible compared to healthy young probands [15, 17, 20]. However, the correlation between aortic dilatation and stiffness was highly significant. Under losartan treatment, a stiffness regression was documented during the first 36 months alongside with a regression in diameter *z*-scores, but this development proved impersistent, adding up to a net progression of stiffness over the entire follow-up period. Although our non-treated MFS cohort showed a mild net stiffness regression over 72 months, both groups presented with an overall comparable development of elasticity. Since Selamet-Tierney et al. stated a significant influence of stiffness on aortic dilation progression [23], we compared our patients with highly increased stiffness levels on equal terms to those with lower ranges. Hereby, we could likewise highlight a tendency of lower AoR progression in patients with less baseline deteriorated elasticity levels. Especially, a significantly lower progression under losartan treatment in those with less stiffened aortic tissue was proven, but again solely during the first 36 months. Therefore, stiffness seems to play a predictive or even an influencing role for medical therapy, but the aortic *z*-score progression after 36 months of losartan treatment was not detained by lower baseline stiffness.

Since the earliest consideration of TGF-ß pathway involvement in MFS pathology [7], clinical and animal trials on ARB outcome were contradictory. Yet, the discrepancy between the high variability in human genetics and only two frequently used MFS mouse models should be taken into consideration. Further, the utilized drug dosages and their application routes were strikingly different. Additionally, the previously administered losartan dosages in clinical trials were empirically chosen and not tested for their circulatory effectiveness. When starting our investigational study in 2009 [15], the intended losartan dosages were based on the proposed 1.0–1.4 mg/kg/day up to 100 mg/day at a maximum. But since indications of better efficacy by using higher dosages have been noticed, we successively augmented the dosages especially in more severely affected patients in recent years. This probably led to the significant correlation of losartan dosage to AoR-involvement, but by comparing the patient subgroups with different degrees of dilatation, statistically similar progression rates of dilatation and elasticity were detectable regardless of initial AoR dimensions. Therefore, losartan dosage could have played a decisive role.

Although the positive correlation of AoR dilation and stiffness data with age was strong in our cohort, no accelerating progression, neither of dilatation nor of elasticity, could be demonstrated by plotting aortic measurements against the respective age of the individual patient. Therefore, a suspected worsening impact of puberty hormones on AoR dilatation did not prove substantial. As it is known from adult MFS patients’ investigations, sex has a significant effect on morbidity and mortality in MFS, as male patients tend to suffer from faster aneurysm progression, earlier surgical interventions and higher aortic dissection rates [24]. In 2000, the membrane-bound angiotensin converting enzyme 2 (ACE2) was discovered as a critical counteracting regulator of Ang-II: ACE2 cleaves Ang-II to the vasodilative enzyme Ang-(1–7), which binds to the Mas receptor [25]. The ACE2-Ang-(1–7)-Mas cascade operates, at least partly, via endothelial nitride oxide synthase activation, therefore being a potential regulator of endothelial cell function [25]. Fluid shear stress and estrogen exert further influence on this pathway [26]. Interestingly, the ACE2 gene is located on the X-chromosome and circulating, inactive ACE2 levels are 50% higher in males [27]. Therefore, a sex influence, either by different gene dosages, polymorphism, or known influences of estrogen, is highly conceivable [26]. Our data confirms the sex differences in MFS regarding greater AoR dilatations in males as early as in childhood. Nevertheless, while presenting with identical age, blood pressure and elasticity levels, both sexes showed comparable progression rates of dilatation and elasticity, and no perceivable acceleration during puberty over the years.

Owing to its observational character, our investigation has its limitations: First, patient numbers are low and their respective follow-up duration is diverse. But since patients were selected by compliance to medication and visits, a further drop-out was non-existent. Additionally, data is lacking interobserver variations since evaluation was done by a single investigator. Second, due to the lack of randomization, the losartan and the no-treatment group did not match, especially in age and cardiac severity. But since a significant severity impact on longitudinal dilation progression was ruled out in the furthermore subdivided losartan groups, the comparison of the former subgroups seemed feasible. Third, the targeted losartan dosage increased within the recent years, especially in the more affected MFS patients. Therefore, comparisons and specific conclusions of this topic were difficult to draw. Last, the variation in genetic pathogenic variants and its impact on phenotype and its possible influence on medical efficacy are ample as already known in this disease, but a specific subdivision by genetic factors seemed impossible due to the sample size and was therefore omitted.

In conclusion, in this-to our knowledge first-longitudinal long-term assessment (> 3 years of follow-up) of a homogenous group of young MFS patients (< 18 years of age) under ARB treatment, a regression of their dilated AoR diameters during the first 3 years was shown, but this effect weakened thereafter was shown. The improvement in aortic dilatation progression by losartan concurred with an amelioration of elasticity. Lower stiffness levels seemed to predict better outcomes overall and superior therapy responses in particular, but only within the first 3 years. But especially in patients with particular high stiffness levels (exceeding the 90th percentile with 7.4 in our cohort), higher aortic progression rates are expected and therefore, specific cut-off levels of elasticity should be included in risked stratifications for these patients. Sex represented a statistically significant influence, identifying higher indexed aortic diameters in males but without affecting AoR progression or tissue integrity over time and without a discernible impact of puberty. Losartan ensured equal AoR progression rates over the entire treatment in all MFS patients regardless of their baseline aortic dilatation severity, although the dosages were significantly augmented in the more affected patients. Therefore, further investigations with larger, randomized trials with follow-up durations of over 3 years should ascertain the encountered decreasing effect of long-term ARB therapy in MFS patients and its possible prevention by higher losartan dosages. Further, the same assessments should be performed for atenolol on equal terms.
